# Penile Replantation in Severe Polytrauma: A Rare Case With Surgical Insights

**DOI:** 10.7759/cureus.112297

**Published:** 2026-07-08

**Authors:** Hector R Gonzalez-Carranza, Seiichi Fuziwara Ruiz, Luis A Reyes-Vallejo

**Affiliations:** 1 Department of Urology, Hospital Angeles Metropolitano, Mexico City, MEX; 2 Urologic Oncology, Centro Médico Nacional Siglo XXI, Mexico City, MEX

**Keywords:** penile amputation, penile replantation, penile trauma, polytrauma patient, reconstructive urology

## Abstract

Penile amputation is a rare urological emergency that requires prompt surgical intervention to restore both anatomical integrity and functional outcomes. Most reported cases are associated with self-inflicted injury, psychiatric disorders, or sharp trauma, whereas high-energy blunt mechanisms are exceedingly uncommon. We present the case of a 29-year-old male who sustained a motor vehicle accident resulting in traumatic right upper extremity amputation and complete penile transection with testicular exposure. Initial life-saving management was performed within the first two hours after injury, while definitive urologic reconstruction was carried out 72 hours after the trauma, corresponding to an estimated ischemia interval of 72 hours before genital reconstruction. Definitive repair included end-to-end anastomosis of the corpora cavernosa and urethra, with preservation of viable tissue and appropriate urinary diversion. Postoperative imaging demonstrated preserved cavernosal integrity and progressive vascular recovery. At the one-month follow-up, a urethral fistula and right testicular infarction were identified, requiring orchiectomy. Ultimately, after three months, the patient achieved complete urethral healing and recovery of urinary function. This case highlights the feasibility of penile reconstruction in the context of severe polytrauma and underscores the importance of multidisciplinary management, early intervention, and careful postoperative monitoring. Given the absence of standardized guidelines, individualized treatment strategies remain essential to optimize outcomes in these complex injuries.

## Introduction

Penile amputation is a rare yet highly challenging injury that requires emergent multidisciplinary surgical intervention and meticulous postoperative care to minimize complications and preserve the patient’s urinary, sexual, cosmetic, and overall quality-of-life outcomes. Etiologies range from psychiatric disorders and self-inflicted injury to traumatic mechanisms, and successful management depends on precise surgical replantation or reconstructive techniques to restore both function and appearance [1,2].

The first successful description of penile reimplantation was published in 1929 by W. S. Ehrich, who reported two cases of direct trauma in which simple approximation of the severed ends allowed satisfactory recovery through the process of wound healing [3]. Since then, more than 118 cases have been reported in the literature, most of them related to sharp trauma or self-inflicted injury [2]. Many self-inflicted cases occur in the context of Klingsor syndrome, which refers to genital self-mutilation most commonly associated with severe psychiatric disorders, psychosis, or religious delusions [4]. However, to our knowledge, no cases secondary to motor vehicle accidents have been described.

From a surgical perspective, complete penile transection is particularly challenging because the penis contains paired corpora cavernosa, the corpus spongiosum, the urethra, and a delicate neurovascular supply. Prolonged ischemia and disruption of these structures may compromise tissue viability, urinary continuity, erectile function, sensation, and cosmetic outcomes, making timely reconstruction and close postoperative monitoring essential [1,2].

We present the case of a 29-year-old patient who sustained a motor vehicle accident resulting in traumatic right upper extremity amputation and complete penile transection with testicular exposure. The objective of this report is to describe the staged surgical reconstruction, postoperative course, and functional outcomes of this rare injury, for which no standardized management guidelines currently exist.

## Case presentation

A 29-year-old male was brought unconscious to the emergency department following a high-energy motor vehicle accident. On admission, the patient presented with severe polytrauma, including traumatic amputation of the right upper extremity and extensive genital trauma characterized by penile degloving, complete proximal penile transection, and testicular exposure. Initial vital signs showed a blood pressure of 98/67 mmHg and a heart rate of 52 beats per minute. His Glasgow Coma Scale score was eight. The patient required transfusion of two units of packed red blood cells during initial resuscitation. Initial trauma imaging showed no evidence of an associated pelvic fracture.

Initial management followed advanced trauma principles and prioritized life-threatening injuries. Within the first two hours after injury, the trauma and orthopedic teams performed emergency surgical management of the right upper extremity amputation, achieving adequate hemorrhage control. During the same initial surgical period, the general surgery team placed a transurethral Foley catheter and performed a cystostomy, followed by wound packing of the genital injury, achieving initial bleeding control.

**Figure 1:**
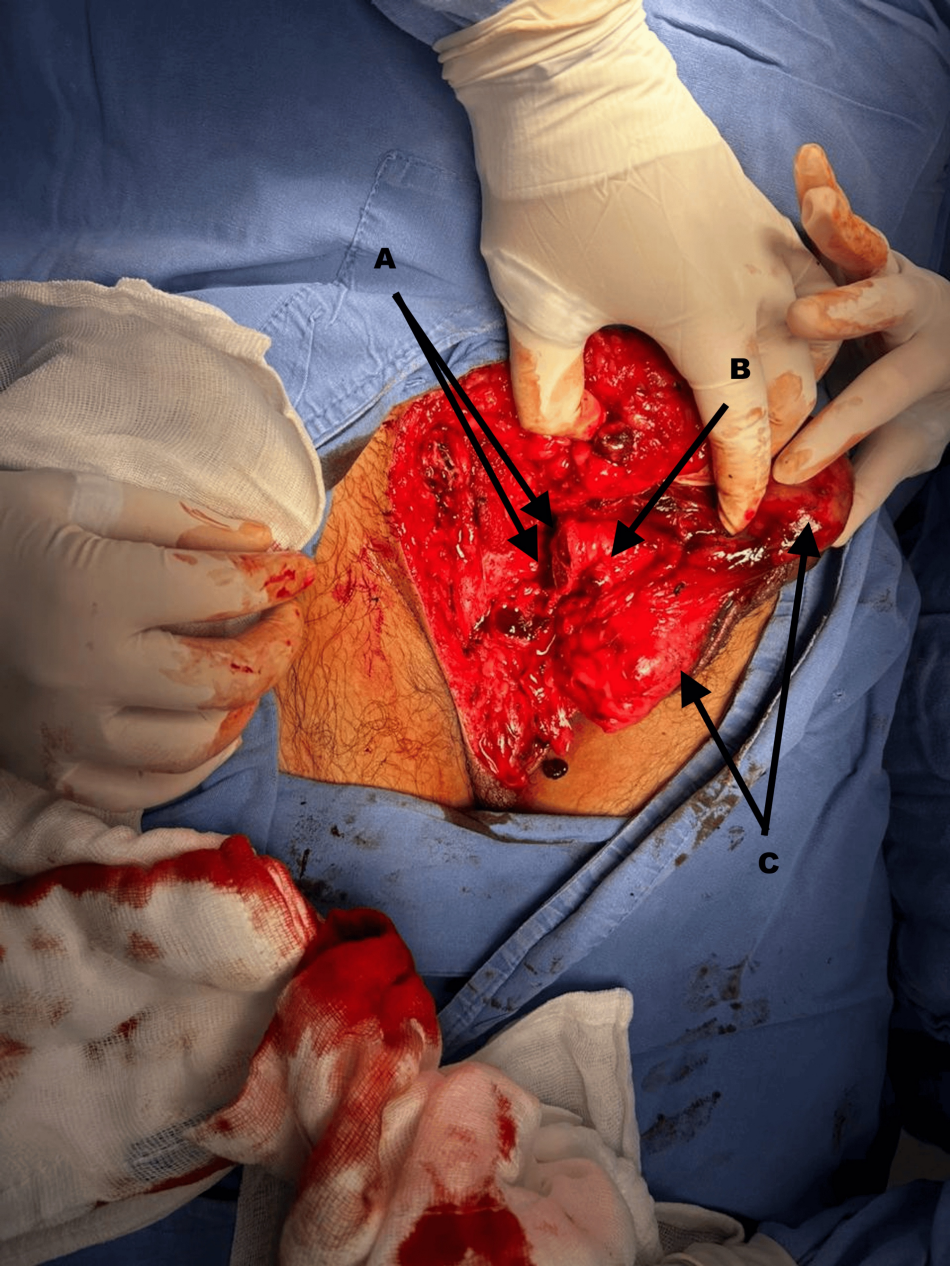
First surgical exploration showing complete transection of the corpora cavernosa, corpus spongiosum, and urethra, with exposed testes A: corpora cavernosa; B: corpus spongiosum and urethra; C: testicles

Forty-eight hours after injury, the patient returned to the operating room for wound irrigation and debridement. During this procedure, complete disruption of both corpora cavernosa, the corpus spongiosum, and the urethra at the proximal penile shaft was identified, prompting consultation with the urology team. Definitive urologic reconstruction was performed 72 hours after injury, corresponding to an estimated ischemia interval of 72 hours before genital reconstruction. The initial intraoperative findings, including complete disruption of the penile erectile structures with exposed testes, are shown in Figure 1.

During definitive surgical exploration, complete disruption of both corpora cavernosa, the corpus spongiosum, and the urethra was confirmed, with total separation of the penile erectile structures. Careful debridement of devitalized tissue edges was performed while preserving as much viable tissue as possible to facilitate anatomical reconstruction.

**Figure 2:**
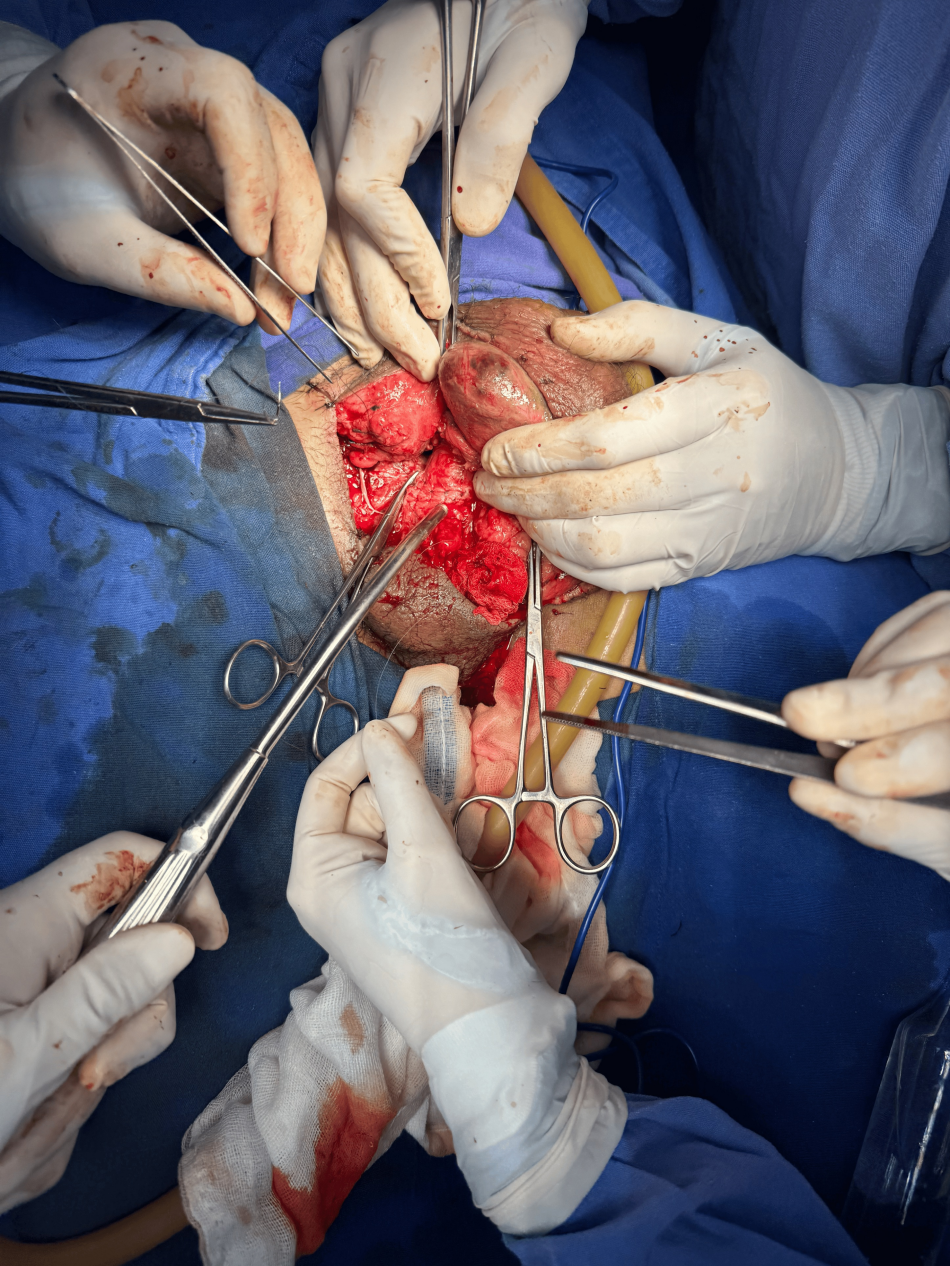
End-to-end anastomosis of the corpora cavernosa, corpus spongiosum, and urethra

Reconstruction of the corpora cavernosa was carried out through end-to-end anastomosis of the tunica albuginea of both corpora cavernosa. Approximation of the edges was achieved using interrupted 4-0 Monocryl® absorbable sutures, restoring anatomical continuity and alignment of the erectile bodies, as shown in Figure 2.

**Figure 3:**
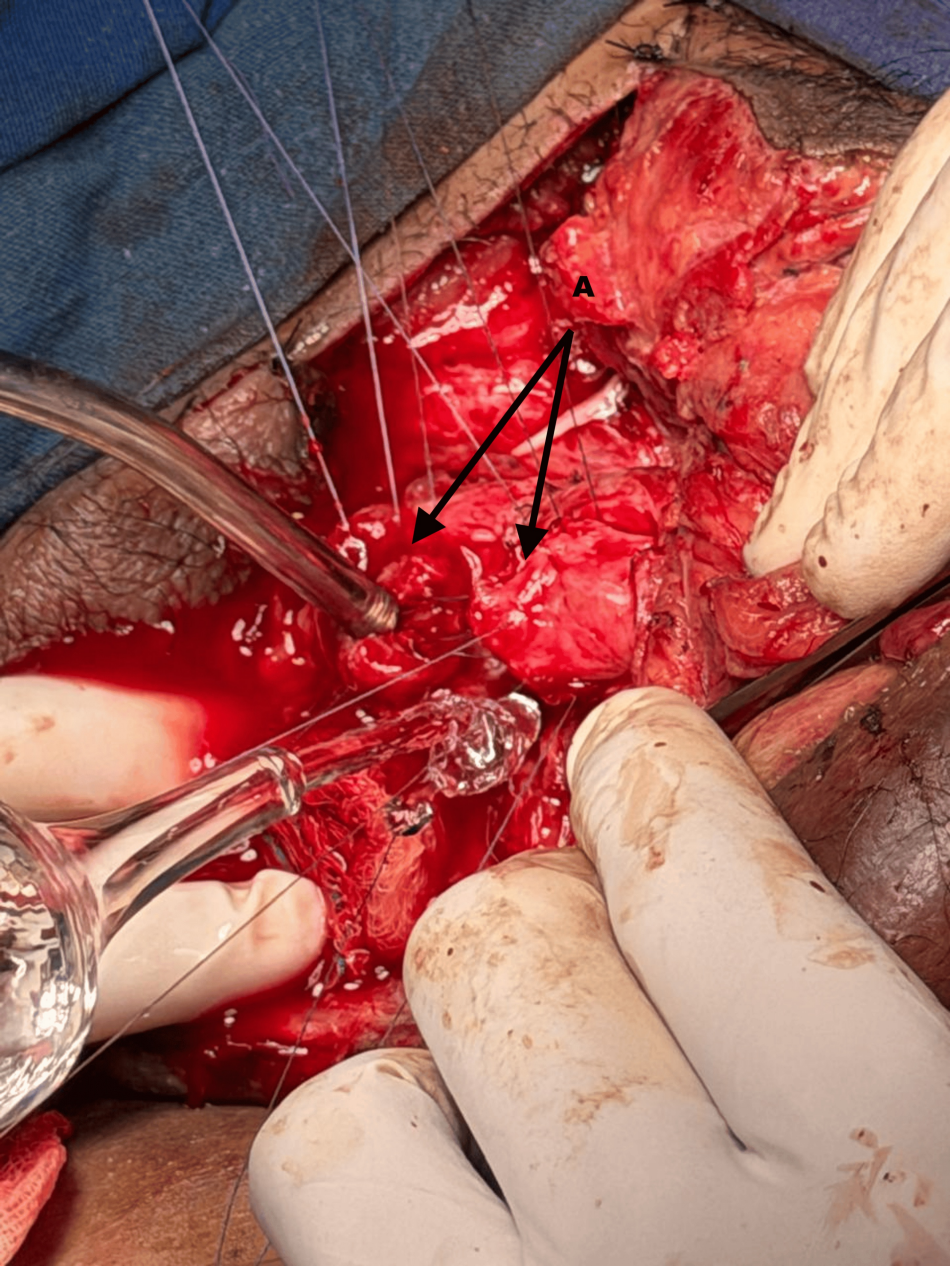
Anastomosis of the corpora cavernosum Intraoperative view demonstrating the transected corpus cavernosum during penile reconstruction. A: transected corpus cavernosum

After restoration of the corporal bodies, the proximal and distal urethral ends were identified. An end-to-end urethral anastomosis was then performed using 4-0 Monocryl® absorbable sutures, ensuring adequate mucosal coaptation and alignment of the surrounding corpus spongiosum. The completed end-to-end reconstruction of the corpora cavernosa, corpus spongiosum, and urethra is shown in Figure 3.

A thorough inspection of both testes and spermatic cords was subsequently performed, revealing no immediate evidence of traumatic injury, vascular compromise, or structural damage. Both testes appeared viable and were returned to the scrotum. Finally, layered closure of the soft tissues was performed, with skin approximation using 2-0 Prolene® sutures. The transurethral Foley catheter and cystostomy were left in place to ensure continuous urinary drainage and protect the urethral anastomosis.

Postoperative management included ertapenem 1 g intravenously every 24 hours for 10 days. Both the cystostomy and transurethral Foley catheter remained in place during the early postoperative period and were removed after one month. The patient remained hospitalized for 10 days.

One week after reconstruction, a penile Doppler ultrasound was performed. The corpora cavernosa and corpus spongiosum demonstrated preserved echogenicity. The right cavernosal artery showed diminished flow at the level of the penile base, whereas the left cavernosal artery and dorsal penile artery demonstrated preserved and hemodynamically stable flow.

One month after surgery, a repeat penile Doppler ultrasound demonstrated improvement in the flow of the right cavernosal artery. The corpora cavernosa and corpus spongiosum continued to show preserved echogenicity. Flow in the left cavernosal and dorsal penile arteries remained normal, and the venous drainage pathways showed no abnormalities.

**Figure 4:**
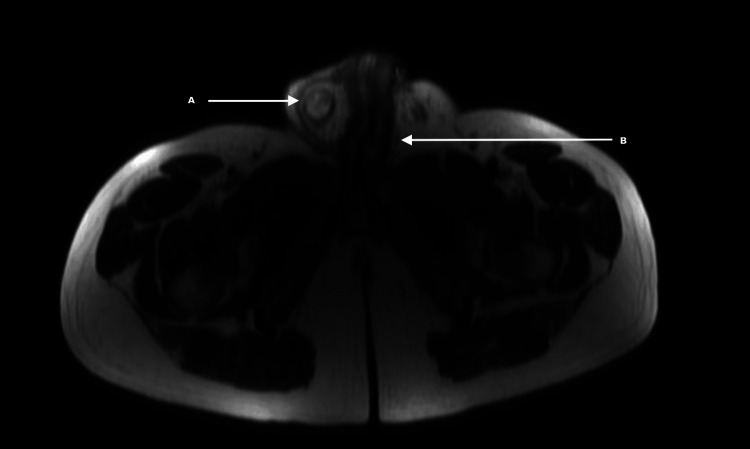
Pelvic magnetic resonance imaging demonstrating postoperative changes at the penile base and right testicular infarction Axial pelvic magnetic resonance imaging demonstrating postoperative changes involving the corpora cavernosa and corpus spongiosum at the proximal penile shaft, penile base, and intracavernosal septum, as well as right testicular infarction. A: right testis with imaging findings consistent with infarction. B: postoperative changes at the penile base

At the one-month follow-up, gadolinium-enhanced pelvic magnetic resonance imaging demonstrated postoperative changes involving the corpora cavernosa and corpus spongiosum at the proximal penile shaft, penile base, and intracavernosal septum. A fistulous tract involving the penile urethra was identified, associated with a 7.3-cc fluid collection in the right scrotal region. Imaging findings were also suggestive of right testicular infarction, as shown in Figure 4; therefore, right orchiectomy was performed without complications.

**Figure 5:**
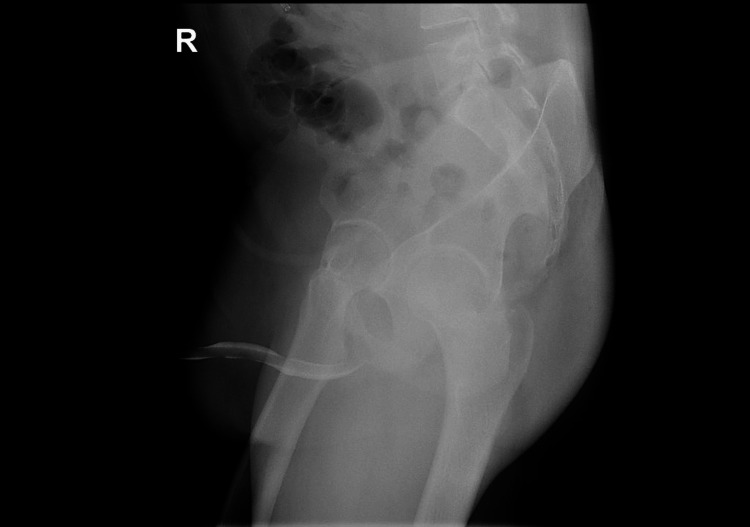
Retrograde urethrography showing no evidence of extravasation

Subsequent retrograde urethrography demonstrated complete urethral healing without evidence of contrast extravasation, as shown in Figure 5, allowing catheter removal. The total follow-up duration was six months. During follow-up, the patient achieved recovery of urinary function; however, erectile function was not preserved, and the patient was unable to achieve erections. Penile prosthesis placement is planned as a future reconstructive intervention.

## Discussion

Penile amputation is a rare and complex urological emergency most commonly associated with psychiatric disorders, self-inflicted injury, or, less frequently, surgical complications. Traumatic penile amputation secondary to blunt mechanisms is exceedingly uncommon. To our knowledge, complete penile transection resulting from a motor vehicle accident, particularly in association with simultaneous upper limb amputation, has not been previously described in the literature.

Raheem et al. reported a comprehensive review of over 118 cases of penile amputation, the majority of which were caused by sharp injuries. In these cases, ischemia times ranged from two to 12 hours, and primary urethral anastomosis combined with corporal reconstruction was the most commonly employed surgical approach. Reported postoperative complications included skin necrosis, infection, urethral strictures, and erectile dysfunction. In contrast, our patient did not develop infectious complications or tissue necrosis, which may be attributed to timely surgical intervention and adequate perioperative management. However, erectile dysfunction remains a concern and may be explained by prolonged ischemia and potential neurovascular injury [2].

More recently, Arbel et al. (2024) analyzed 46 cases of penile replantation from multiple countries and found that voiding function was preserved in 91.3% of patients. This is consistent with our findings, as our patient achieved complete recovery of urinary function following catheter removal. These data support the notion that, even in severe cases, restoration of urinary function is achievable when appropriate surgical techniques are applied [4].

A key distinguishing feature of our case is the mechanism of injury. Unlike most reported cases involving only sharp trauma, this injury resulted from both sharp and high-energy blunt force, which likely caused more extensive soft tissue damage, vascular compromise, and associated systemic injuries. This significantly increased the complexity of management and may negatively impact functional outcomes, particularly regarding erectile function [5,6].

Management of such cases requires strict adherence to trauma principles, prioritizing life-threatening injuries before proceeding to reconstructive efforts. In polytrauma patients, the timing of genital reconstruction must be carefully balanced against overall hemodynamic stability. Early surgical intervention remains critical to minimize ischemia time and optimize tissue viability [7,8].

Functional outcomes following penile replantation are variable and depend largely on ischemia duration, preservation of neurovascular structures, and surgical technique. While urinary outcomes are generally favorable, erectile function is less predictable and may be compromised due to endothelial damage, corporal fibrosis, or nerve injury. Long-term follow-up is essential to fully assess functional recovery and the potential need for additional interventions [9].

Given the rarity of penile amputation, particularly in the context of high-energy polytrauma, there are no standardized management guidelines. As a result, treatment strategies rely heavily on surgical expertise, available resources, and individualized patient assessment.

This case highlights the feasibility of genital reconstruction even in the setting of severe multisystem trauma and underscores the importance of early multidisciplinary management to optimize both functional and anatomical outcomes.

## Conclusions

Penile amputation secondary to high-energy blunt and sharp trauma is an exceedingly rare entity, especially when occurring in the context of severe polytrauma. This case highlights that successful anatomical reconstruction and preservation of urinary function are achievable with prompt multidisciplinary intervention. However, functional outcomes such as erectile function remain variable and are influenced by ischemia time and neurovascular integrity. In the absence of established guidelines, management should be individualized, emphasizing early intervention and coordinated care.
